# Intensification of Vero cell adherence to microcarrier particles during cultivation in a wave bioreactor

**DOI:** 10.3389/fbioe.2025.1542060

**Published:** 2025-02-14

**Authors:** Z. K. Mazhed, V. E. Vasilenko, A. A. Siniugina, K. V. Kaa, A. S. Motov, K. O. Pokidova, Y. Y. Ivin, A. N. Piniaeva, Y. K. Khapchaev, K. A. Chernov, A. A. Ishmukhametov

**Affiliations:** ^1^ Federal State Autonomous Scientific Institution “Chumakov Federal Scientific Center for Research and Development of Immune and Biological Products of the Russian Academy of Sciences” (FSASI “Chumakov FSC R&D IBP RAS”), Moscow, Russia; ^2^ Institute for Translational Medicine and Biotechnology, First Moscow State Medical University (Sechenov University), Moscow, Russia

**Keywords:** cell cultivation, microcarrier particles, bioreactor technology, Vero cells, wave bioreactor, cytodex-1, vaccine production, upstream process development

## Abstract

Vaccination is the most effective strategy for fighting viral diseases, with both live and inactivated vaccines remaining crucial despite advancements in subunit vaccine technologies. A key player in vaccine production is the Vero cell line, derived from the kidney cells of the African green monkey, which is essential for manufacturing vaccines against diseases like polio, rabies, yellow fever, and COVID-19. The efficiency of Vero cell cultivation directly impacts vaccine production, often utilizing bioreactors ranging from small (1–10 L) to large (up to several thousand liters). Wave-type bioreactors are commonly employed for initial cell propagation due to their simplicity. However, achieving uniform cell distribution on microcarriers in these systems poses challenges. This study aims to evaluate intermittent stirring during the early cultivation stages to enhance Vero cell distribution and growth, potentially improving overall cultivation efficiency.

## 1 Introduction

Vaccination remains the most effective method of combating viral diseases. Despite the rapid development of subunit vaccine technologies, the well-studied methods for manufacture of live and inactivated vaccines remain relevant and widely used. The industrial manufacture of such vaccines is carried out through the cultivation of viral pathogens in a producing culture. One of the most widely utilized cultures is (Yang et al., 2019a), Vero–an adhesive cell line obtained from the epithelial kidney cells of the African green monkey (Chlorocebus aethiops) (Kiesslich and Kamen, 2020). It is used to manufacture a large number of key vaccines against diseases such as polio (Piniaeva et al., 2021), rabies (Montagnon, 1989), tick-borne encephalitis (Vorovitch et al., 2020), yellow fever (Pato et al., 2019), COVID-19 (Kozlovskaya et al., 2021), and chikungunya fever (Tiwari et al., 2009).

One of the questions that arise before modern biotechnology is how to improve the efficiency of industrial methods for Vero cells cultivation, thus, increasing manufacturing performance for many vaccines. To obtain a Vero cell culture for further virus infection, a series of bioreactors are used; the last one, in which the cells are infected and the viral material is harvested, can have a volume between 50 and several thousand liters. The first bioreactor in this line may have a working volume of only 1–10 L, but the efficiency of the entire process may depend on the performance of the initial stage (Bakker et al., 2011). A wave-type bioreactor (e.g., ReadyToProcess Wave 25, Biostat™ RM, etc.) is often used during the initial stage of Vero cell propagation because of simplicity and accessibility. Moreover, bioreactors of this type are commonly utilized in the development of novel drugs and in laboratory experiments (Thomassen et al., 2012).

Adhesive cell cultures, including Vero, are mostly cultured in stirred bioreactors using microcarriers. The efficiency of this process depends on multiple factors, including mixing speed, microcarrier type and material, serum concentration, etc. One of the key factors is the uniform distribution of cells on the microcarrier surface, as uneven distribution may result in empty microcarrier particles and, as a consequence, to a decrease in the final cell concentration (Derakhti et al., 2019). This problem is especially challenging if cells are propagated in wave bioreactors, in which a thin layer of growth medium with cells and microcarrier is mixed by waves that arise from the cyclic movement of the bioreactor platform (Eibl et al., 2009). Microcarrier and cells have different sedimentation coefficients (Satoh et al., 1991). Therefore, at the early stage of cultivation, it is quite difficult to select the parameters of the wave-mixed bioreactor platform movement (rocking angle, rocking rate, angular velocity), at which the cells and microcarrier will be distributed evenly in the suspension (Thomassen et al., 2012).

One way to improve the uniformity of cell attachment is the intermittent stirring (Ng et al., 1996). The intermittent stirring at the initial stage of cultivation allows the microcarrier and the cells to settle at the bottom of the bioreactor vessel, thus, simplifying the process of cell attachment. The aim of the study was to assess the applicability of this method for increasing the uniformity of distribution and growth of Vero cells during cultivation in a wave bioreactor, as well as to investigate the effect of this method on further stages of cell line cultivation.

## 2 Materials and methods

### 2.1 Cell culture

The Vero WHO RCB 10-87 cell line (passages 141–149) was used in the study.

### 2.2 Cell cultivation in roller bottles

Vero cells, used for inoculation of the wave bioreactor, were propagated in an incubator at 37°C, in 850-cm^2^ roller bottles (Corning™) containing Eagle’s MEM medium with Hanks’ Balanced Salt Solution (Chumakov FSC R&D IBP RAS” (Institute of Poliomyelitis), Russia), 10% fetal bovine serum (BioloT, Russia), and 1% gentamicin. The cells were removed from the growth surface using Versene solution (0.02%, Institute of Poliomyelitis, Russia) and trypsin solution (0.25%w/v, Institute of Poliomyelitis, Russia).

### 2.3 Microcarrier preparation

The Cytodex 1™ microcarrier was hydrated according to the manufacturer’s instructions: the required amount of microcarrier was saturated with phosphate buffer solution, using 3 g of microcarrier per 1 L of solution. The microcarrier was thoroughly mixed to ensure uniform distribution of moisture over its surface. The microcarrier suspension was sterilized by autoclaving at t = 121°C for 30 min.

### 2.4 Standard Vero cells cultivation in wave bioreactor

**FIGURE 1:**
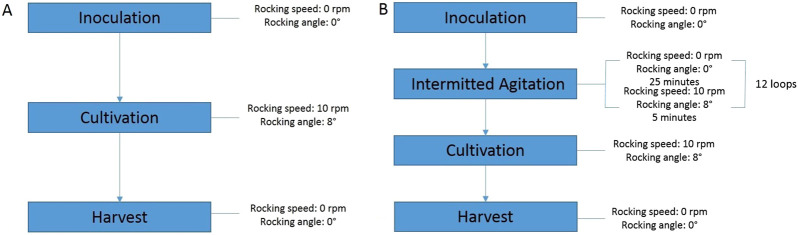
Schematic of rocking speed and rocking angle settings during cultivation on the ReadyToProcess Wave 25 wave bioreactor **(A)** - Under standard conditions, **(B)** - Using “Program”.

A ReadyToProcess WAVE 25 wave bioreactor system (Cytiva, United States) was used to cultivate Vero cells on microcarrier particles. The bioreactor bag (10–25 L working volume, equipped with disposable optical pH and DO sensors), containing MEM (Institute of Poliomyelitis, Russia) with 10% fetal bovine serum (Biolot, Russia) and 3 g/L Cytodex 1™ microcarrier (Cytiva, United States) was inoculated with Vero cells at a density of (1.8 ± 0.5) * 10^5^ cells/mL. The total cultivation volume was 10 L. The dissolved oxygen (DO), temperature, and pH parameters were set to 70%, 37°C, and 7.3, respectively. The content of the bioreactor bag was rocked at 10 rpm with an 8° of rocking angle for 96 h (Figure 1A). The bioreactor bag was supplied with gas mixture (oxygen, CO_2_, and air) at 0.3 L/min pH was controlled by infeed of CO_2_ and 7.5% sodium bicarbonate solution using a peristaltic pump. The DO parameter was controlled by feeding of compressed air or oxygen. Wave bioreactor settings at the intermittent agitation stage.

### 2.5 Vero cells cultivation in wave bioreactor with “program”

The settings of the wave bioreactor using intermittent agitation were identical to the standard one, however, after inoculation, the bioreactor produced 12 cycles of agitation stops within 6 h, where one cycle includes 25 min of stopping in the horizontal position, and 5 min of agitation. Experimentally, we found that 25 min was sufficient for settling of the microcarrier and cells, and 5 min for complete agitation (Figure 1B). A program for intensifying cell attachment, consisting of a sequence of steps, was composed using Unicorn™ software (Cytiva, United States).

### 2.6 Cell cultivation in a disposable 50L bioreactor with vertical mixing

The cells obtained in the wave bioreactor were used to cultivate Vero cells in a 50L stirred bioreactor (STR50, Sartorius Stedim™). The microcarrier with attached cells was allowed to settle in the Wave bioreactor bag, then the growth medium was removed and the microcarrier was treated with Versene solution. The cells were dispersed by incubation in a 0.15% trypsin solution and transferred into a prepared STR50 bag containing pre-heated Eagle’s MEM medium with 7.5% fetal bovine serum and 3 g/L Cytodex 1™ microcarrier. The cells were cultivated for 4 days using the following parameters: 37°С, 50% DO, pH 7.3, 30 rpm.

### 2.7 Assessment of the state of cells on microcarrier particles and calculation of cell con-centration

In order to assess the state of the cell growth and to calculate the cell concentration, daily samples were taken from the bioreactor bag. The state of the cell growth was assessed visually using an inverted microscope. The cell concentration was determined by staining cell nuclei with 0.1 M citric acid containing 1% crystal violet and counting the nuclei in a hemocytometer.

### 2.8 Statistical analysis

Statistical analysis was performed using 2-way ANOVA and the Shapiro-Wilk test using GraphPad Prism 8.0 software.

## 3 Results

**FIGURE 2:**
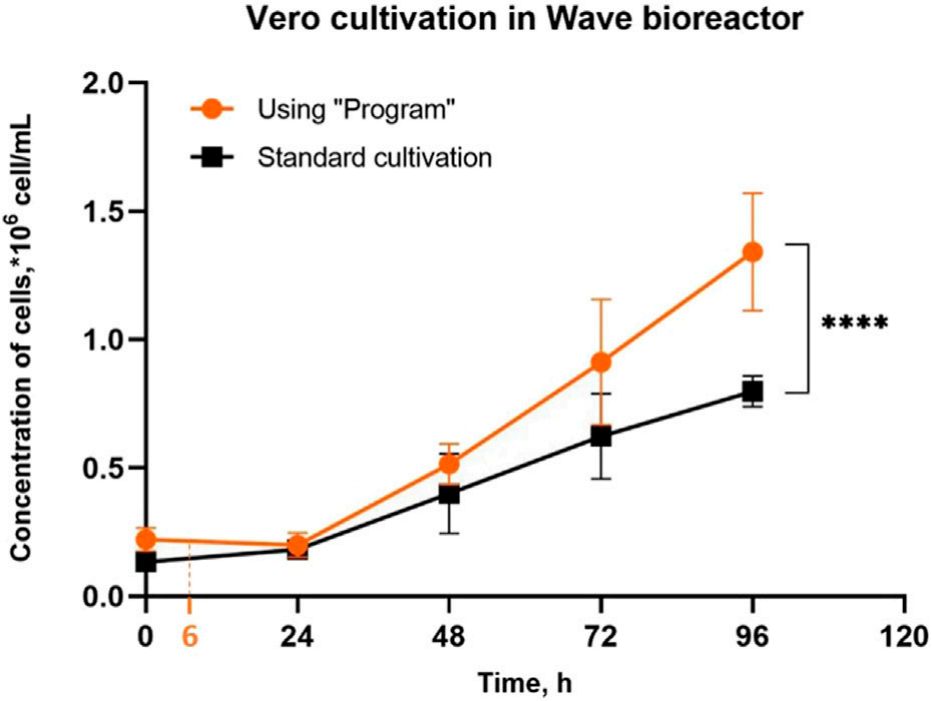
Vero cell growth in a ReadyToProcess WAVE 25 bioreactor on Cytodex 1™ microcarrier using the “Program” and during the standard cultivation. Intermitted agitation includes 12 cycles (cultivation with Program, Figure 1B) and takes 6 h. Experiments were performed in twelve replicates; the data are presented as mean and standard deviation. The experimental data were analyzed using the 2-way ANOVA, **** - p-value <0.0001.

**FIGURE 3:**
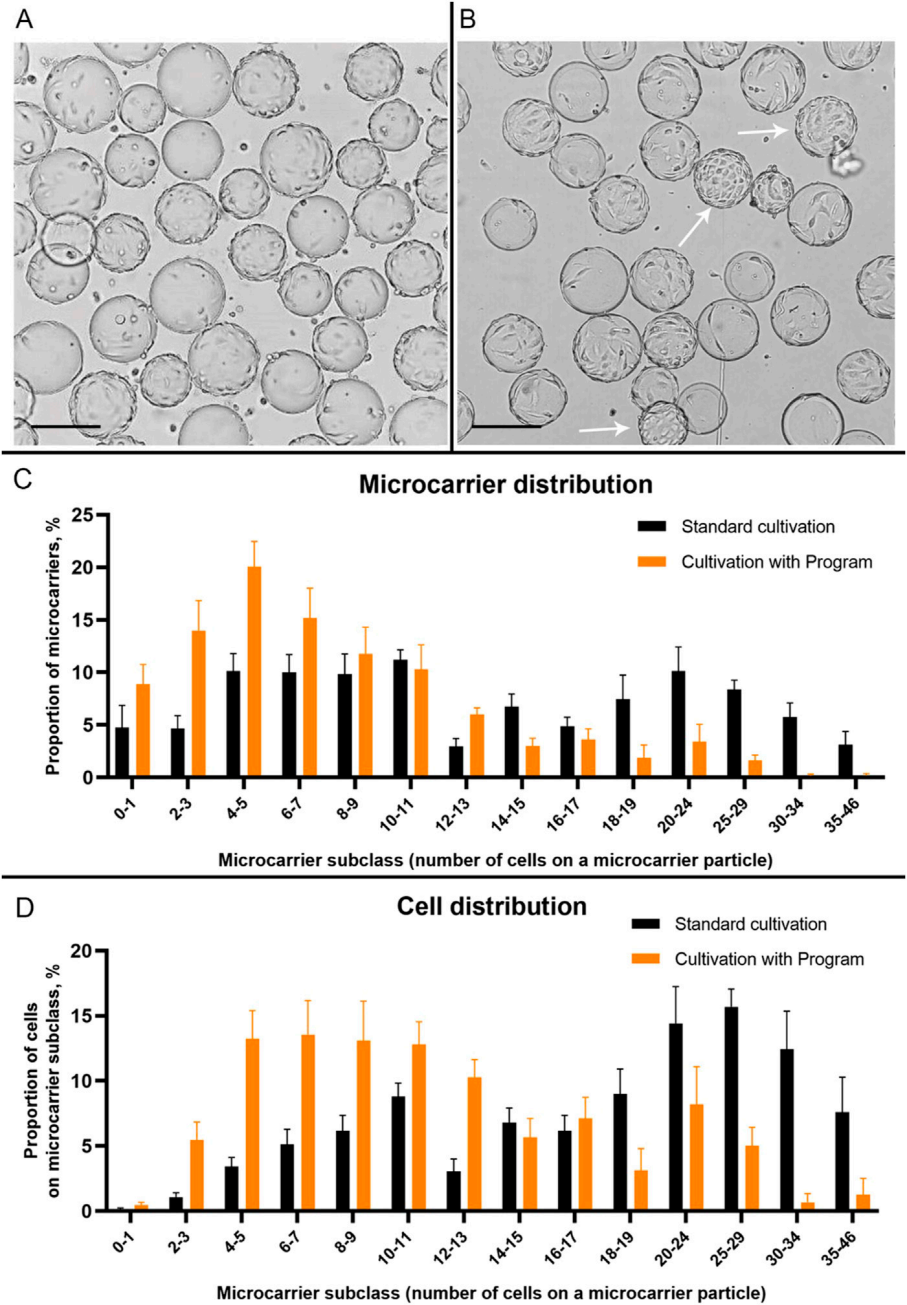
The condition of the Vero cell monolayer on Cytodex 1 microcarrier particles at 24 h after the start of cultivation in a Wave bioreactor using the “Program” **(A)** and standard cultivation conditions **(B)**. Arrows indicate microcarrier particles with a dense monolayer of Vero cells. Scale bars corresponds to 200 µm. **(C)** Distribution of microcarriers Cytodex 1 by subclasses depending on their coverage with Vero cells 24 h after inoculation in a Wave bioreactor during the standard cultivation and cultivation with “Program”. **(D)** Distribution of Vero cells on different subclasses of Cytodex 1 microcarrier 24 h after inoculation in a Wave bioreactor during the standard cultivation and cultivation with “Program”. To construct graphs, 18 images of microcarrier suspensions from different experiments were used (9 images for a standard cultivation and 9 - for the cultivation with Program).

Following a series of experiments aimed at growing Vero cells on Cytodex 1™ microcarriers in a wave bioreactor, we found that, while the same cell concentration was used for inoculation, the final concentration was higher when using the “Program” (Figure 2). During each cultivation process, samples of the microcarrier suspension were analyzed in terms of cell concentration and inspected visually using a light microscope. We have found that in both cases (Standard and Program cultivation) cells were attached to the microcarrier after 24 h post inoculation. However, no particles in the suspension completely covered with cells were observed (Figure 3A). At the same time, under standard cultivation conditions, some microcarrier particles are already covered with a dense layer of cells (Figure 3B). The results of visual examination demonstrate that under standard cultivation conditions (i.e., without using the “Program”), Vero cells are unevenly distributed between the microcarrier particles: too many cells bind to some particles, forming a dense monolayer after 24 h of cultivation.

For statistical processing of images of Vero cells attached to microcarriers 24 h after inoculation, we divided the microcarrier particles into subclasses based on the number of visible cells on the particles (Figures 3C, D). We found that when using the Program, the vast majority of microcarriers after 24 h have from 2 to 13 cells on their surface (77.3% ± 7.8%) and very few microcarriers are completely covered with cells (from 20 to 46 cells per particle): 5.4% ± 2.7%. At the same time, using standard cultivation, only 48.2% ± 5.2% of microcarriers have from 2 to 13 cells on their surface, and filled microcarriers (from 20 to 46 cells) make up 27.4 ± 5.0 of the total number (Figure 3C). Thus, during standard cultivation, we observed a large proportion of particles on which cell growth was no longer possible, which reduced the overall efficiency of the process of culturing the pseudo-suspension cell culture.

In addition, more than half of the introduced cells (50.2% ± 7.9%) during standard cultivation were on filled microcarriers (from 20 to 46 cells per microcarrier, Figure 3D). This type of cell distribution limits these cells to achieve high final concentrations, already shown on the growth curve (Figure 2). When using the Program, most of the introduced cells (68.5% ± 6.8%) were located on microcarriers with a high potential for covering their surface (from 2 to 13 cells per microcarrier) 24 h after inoculation (Figure 3D).

**FIGURE 4:**
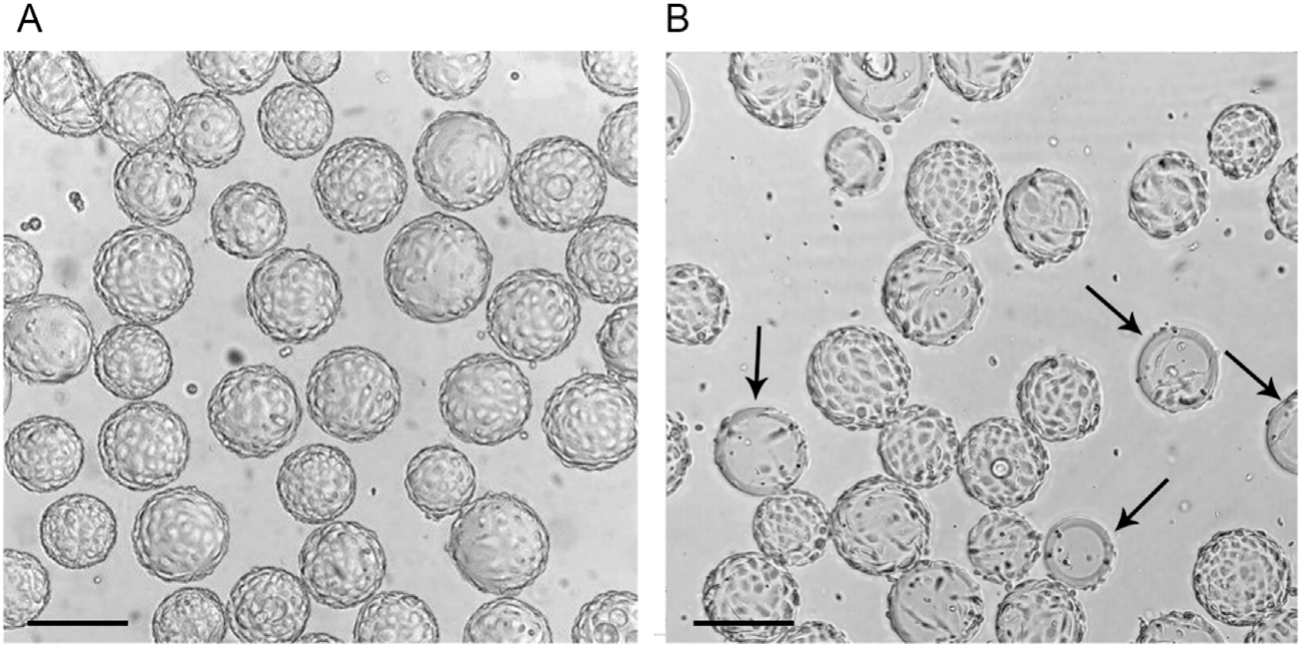
The condition of the Vero cell monolayer on Cytodex 1 microcarrier particles 72 h after the start of cultivation in a Wave bioreactor using the “Program” **(A)** and standard cultivation conditions **(B)**. Arrows indicate microcarrier particles with a large surface area not covered by cells. Scale bars corresponds to 200 µm.

At a later stage of Vero cells cultivation in a wave bioreactor using the “Program”, the evenly covered microcarrier particles were observed (Figure 4A). Under standard conditions, we identified another sign of uneven growth of Vero cells on microcarrier particles: some particles remain not covered (Figure 4B). The high uniformity of Vero cell adherence to microcarrier particles when using the “Program” predetermined the uniformity of subsequent cell growth, which ultimately led to a higher final cell concentration compared to standard conditions.

**FIGURE 5:**
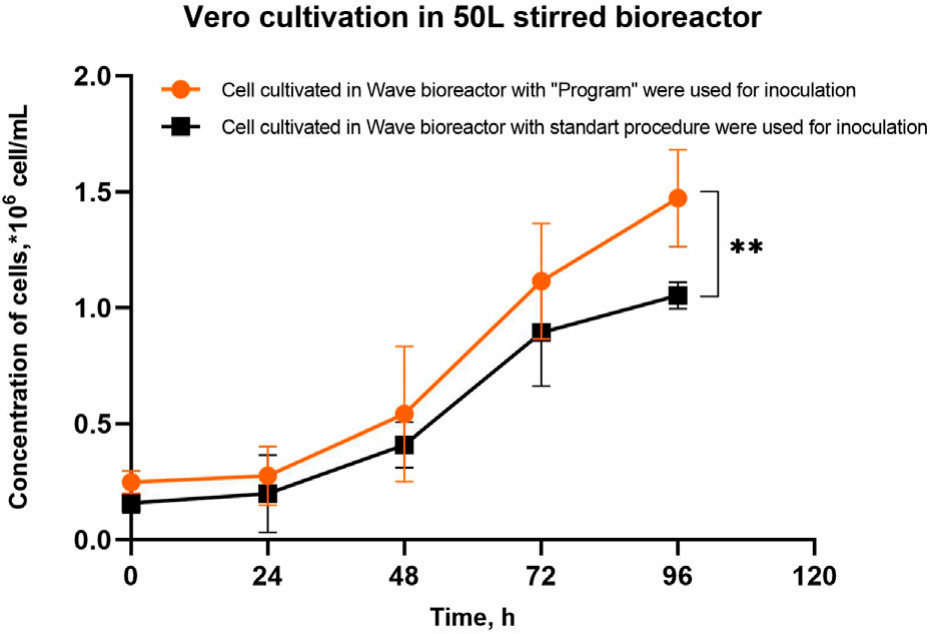
Vero cell growth in a 50L STR50 bioreactor with vertical mixing on Cytodex 1™ microcarrier particles inoculated with cells obtained in the Wave bioreactor with using the “Program” and standard conditions of cultivation. Experiments were performed in six replicates; the data are presented as mean and standard deviation. The experimental data were analyzed using the 2-way ANOVA, ** - p-value <0.01.

The Vero cells obtained in the wave bioreactor under the “Program” and standard conditions were used to inoculate a 50L stirred bioreactor. It was found that when using cell harvests obtained in the wave bioreactor under the “Program” conditions, the final cell concentration in the 50-L bioreactor was higher than when using Vero cells harvested from Wave 25 under standard conditions (Figure 5). We have shown that intensification of Vero cell adherence to microcarrier particles in a wave bioreactor has a positive effect on the efficiency of cell cultivation of both the initial and the later stages of Vero propagation.

## 4 Discussion

Because of its sensitivity to multiple viral pathogens, Vero cell line remains one of the most important producing cultures used in manufacture of a number of viral vaccines (Piniaeva et al., 2021; Kozlovskaya et al., 2021; Kaa et al., 2023). Being a substrate-dependent cell line, Vero is grown on an industrial scale as a pseudo-suspension using microcarrier particles (Yang et al., 2019b). Despite the fact that the technology has been known for several decades, numerous studies are being conducted to improve the efficiency of the Vero cell line growing on microcarrier (VAN WEZEL, 1967; Sun et al., 2004). One of the ways to improve this technology is to increase the uniformity of cell distribution throughout the particles.

To address this problem in the framework of utilizing Wave bioreactors for propagation of Vero cells, at the initial stage of the cultivation process we utilized the intermittent stirring: several cycles of stirring alternate with a pause-sedimentation stage. During the pause, the microcarrier particles and the cells settle to the bottom of the bioreactor vessel and bind together, and during the stirring stage, the free cells and microcarrier particles are mixed in order to settle uniformly during the next pause.

We have shown that this approach increases the uniformity of cell distribution throughout the microcarrier particles (Figures 3A, 4, 5) and consequently rises the final concentration of cells in the bioreactor (Figure 2). Under standard conditions, at the initial stage, the content of the bioreactor is stirred continuously and the cells adhere to the surface of the microcarrier. Due to the difference in the density of microcarrier particles and individual cells, their effective mixing requires labor-intensive research to select optimal mixing conditions for each type of microcarrier, cell type, bag shape and volume. Without such studies, it is likely that most of the heavier microcarrier suspension, together with cell conglomerates, locates near the bottom of the bioreactor bag, while lighter individual cells distributes throughout the volume. Under such conditions, it is difficult to expect a uniform distribution of cells over the surface of the microcarrier spheres, which was confirmed in the present study (Figure 4B). Theoretically, one of the easiest ways to obtain a uniform suspension would be to increase the rate of rocking and/or rocking angle. However, this may lead to an intensificating of the foaming process and to an increasing negative physical impact on the cells, which can negatively affect their viability. For this, as we have already mentioned, a more painstaking and time-consuming selection of conditions is required. It should be noted that after the cell adhesion to microcarrier particles, only one type of particles is present in the suspension–spheres with cells on the surface. Therefore, the problem of uniform mixing is not a major concern.

Intensification of cell adherence to the microcarrier in a wave bioreactor (Stage 1 of cell massive production) leads to an increase in the productivity of Stage 2 – the growth of Vero cells in a 50-L bioreactor (Figure 5). The proposed method of cultivating Vero cells allows for increased performance of the entire potential production line.

This Wave bioreactor culture approach can be applied not only to Vero, but also to other adhesive cell lines (MRC-5, BHK-21, etc.) that are used to produce vaccines for humans and animals. An additional selection of cultivation parameters might be required for other cells; however, the use of this approach to intensifying cell adherence to the surface of microcarrier particles seems to be universal.

This method can also be used in other technologies for culturing adherent cell cultures using microcarriers (e.g., spinner flasks). However, it will be necessary to experimentally select the necessary stirring speeds and stirring stop times depending on the volume of the bioreactor. In our study, rocking speed and rocking angle were selected based on scientific articles (Thomassen et al., 2012), and applied with some modifications. This allowed to reduce foaming and to obtain stable and repeatable results. The stop time and angle were selected experimentally based on the working volume of the bioreactor bag and the actual volume used during cultivation.

## 5 Conclusion

We have successfully developed a program using Unicorn™ software (Cytiva, United States), which allows us to achieve a more uniform distribution of cells. This increases the final concentration of Vero cells when cultured on a ReadyToProcess Wave 25 bioreactor (Cytiva, United States), which is important in the production of vaccines against diseases like polio, rabies, yellow fever, and COVID-19.

## Data Availability

The original contributions presented in the study are included in the article/supplementary material, further inquiries can be directed to the corresponding author.
